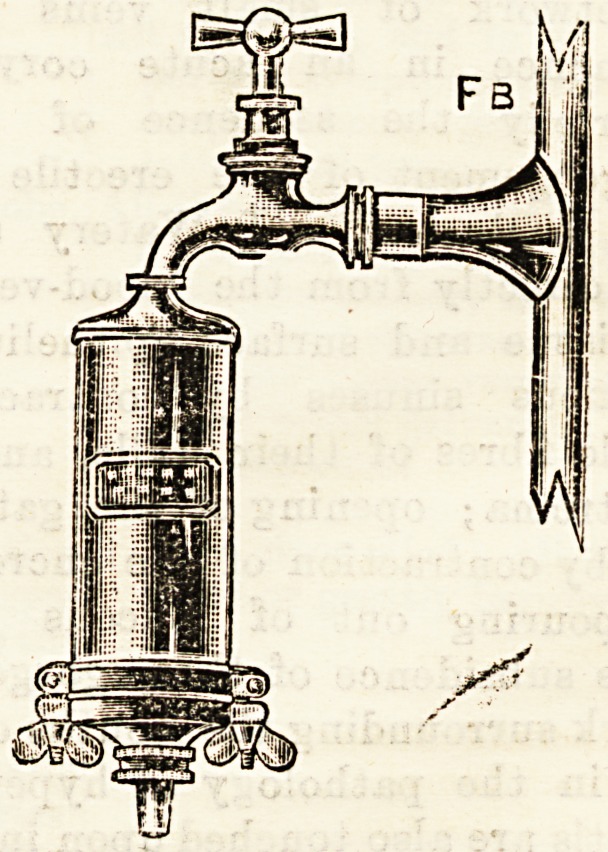# New Appliances and Things Medical

**Published:** 1895-11-02

**Authors:** 


### NEW APPLIANCES AND THINGS MEDICAL.

[We shall be glad to receive, at our Office, 428, Strand, London, W.C., from the manufacturers, specimens of all new preparations and appliances which may be brought out from time to time.]

### THE BERKEFELD FILTER.

(The Berkefeld Filter Company (Limited), 121, Oxford Street, London. W.)

We have received from the Berkefeld Filter Company specimens of their latest types of pressure and non-pressure filters, and have submitted the same to our expert for examination and report. It will be recollected that last year Drs. Sims Woodhead and Cartwright Wood were commissioned by one of our contemporaries to inquire into the efficiency of the domestic filters in use in this country, and that, although their report was never completed, they adduced sufficient evidence to show that, with the exception of the Pasteur-Chamberland filter and the filter supplied by this company, they all failed in retaining pathogenic and non-pathogenic organisms present in the unfiltered water. In their test of the Berkefeld filter it was found that when staphylococcus pyogenes aureus, yeasts, cholera bacillus, and typhoid bacillus, were present in the unfiltered water to the extent of from 3,000—10,000 per cubic centimetre, no organisms appeared in the filtrate for a period of four days. It is therefore evident that under the conditions of these experiments the Berkefeld filter delivered sterile water for that period. Dr. Percy Frankland similarly obtained a sterile filtrate from two new Berkefeld filters in 1893 after twenty minutes' action.

Dr. Plagge, in Germany, has also made a prolonged study of these Kieselguhr filters, and has obtained very satisfactory results, and concludes that the Kieselguhr filter represents the greatest step forward in the science of filtration during the last ten years since they supply in a given time five to ten times the quantity of water yielded by a Pasteur-Chamberland filter, and yield an absolutely sterile filtrate. A filter which supplies a quarter to half a gallon per minute is one which will be tolerated by the domestic servant, whereas filters yielding a slow filtrate have a serious objection in practical use. It must not, however, be forgotten that the slowness of the Pasteur filter can readily be overcome by using a battery of several candles, so that the objection raised by Dr. Plagge as to its practical use is not a serious one if the question of cost does not enter into the problem. The Berkefeld filters also, although having initially a very high rate of flow, soon diminish in their yield, and Dr. Percy Frankland has shown that within the first half-hour of their being in continuous use the rate of filtration in two cases was roughly reduced by one-half. With Glasgow water, which comes from a very pure source, the same observer found that a new filter was brought to almost a complete standstill within twenty-four hours, and our own experiments with London water have given similar results. A more serious objection to the use of the Berkefeld filter is the fact that, as shown by Dr. Johnson in Edinburgh, and Dr. Plagge himself in his numerous experiments, the organisms in the unfiltered water can after a lapse of some time grow through the Kieselguhr filtering surface, and so eventually contaminate the filtered water. Experiments on this point have been made by many observers, but it is important to recollect that even the Pasteur-Chamberland candle permits of a similar growth through the filtering medium after prolonged use. It must also be borne in mind that pathogenic organisms do not retain their vitality in certain waters, and that, therefore, if the water does not afford a suitable pabulum for their normal growth, the absence of organisms in the filtered water may be attributed to this cause, and not to the efficiency of the filtering medium itself. Some such explanation as this must be necessary in order to harmonise the satisfactory results obtained by Drs. Sims Woodhead and Cartwright Wood, and the less favourable results obtained by Dr. Plagge, who, although strongly in favour of the Berkefeld filter, admits the possibility of growth through the walls of the candle with waters which are suitable for the development of the organisms, and contamination of the filtrate on the second day. He therefore advocates that the directions for use ought to insist that for domestic filtration at least two cylinders should be purchased, and that these should be changed every day. The cylinder used the day before would then be boiled and cleansed during the day, and left in the boiling pan well covered ready for use the following morning. Under these conditions the Berkefeld filter appears to be as safe to use as the Pasteur-Chamberland form, and the question resolves itself at the present day into a selection between a battery of Pasteur filters involving only occasional cleansing, but high initial cost to that of a couple of Berkefeld filters obtainable at a more moderate sum but necessitating a daily alternate sterilising as recommended by Dr. Plagge. The Kieselguhr filters have to be handled with considerable care, as they are far more fragile than those made of porcelain. We have satisfied ourselves that both the pressure and non-pressure filters, supplied by this company when previously sterilised, yield water free from organisms when first put into action.

## Figures and Tables

**Figure f1:**
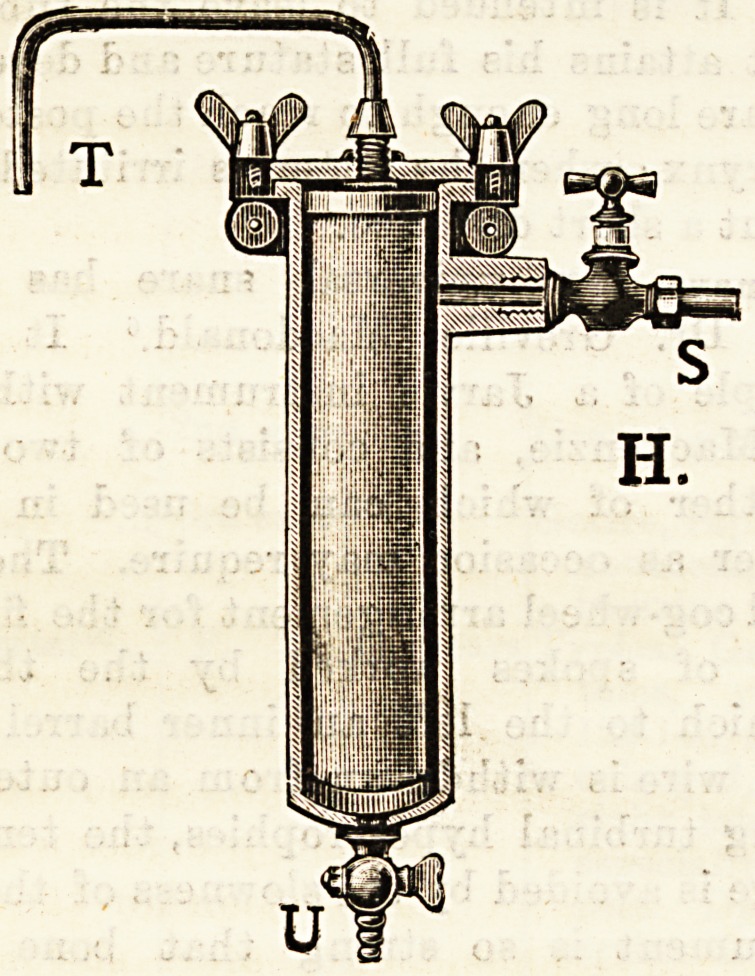


**Figure f2:**